# Exploring Novel Antibiotics by Targeting the GroEL/GroES Chaperonin System

**DOI:** 10.1021/acsptsci.4c00397

**Published:** 2024-12-11

**Authors:** Yuming Wang, Zhou Tong, Jingchun Han, Chuangchuang Li, Xiuping Chen

**Affiliations:** † State Key Laboratory of Quality Research in Chinese Medicine, Institute of Chinese Medical Sciences, University of Macau, Macao 999078, China; ‡ Shenzhen Grubbs Institute, Department of Chemistry, Southern University of Science and Technology, Shenzhen 518055, China

**Keywords:** Infectious diseases, GroEL/ES, Antibiotics, Drug discovery

## Abstract

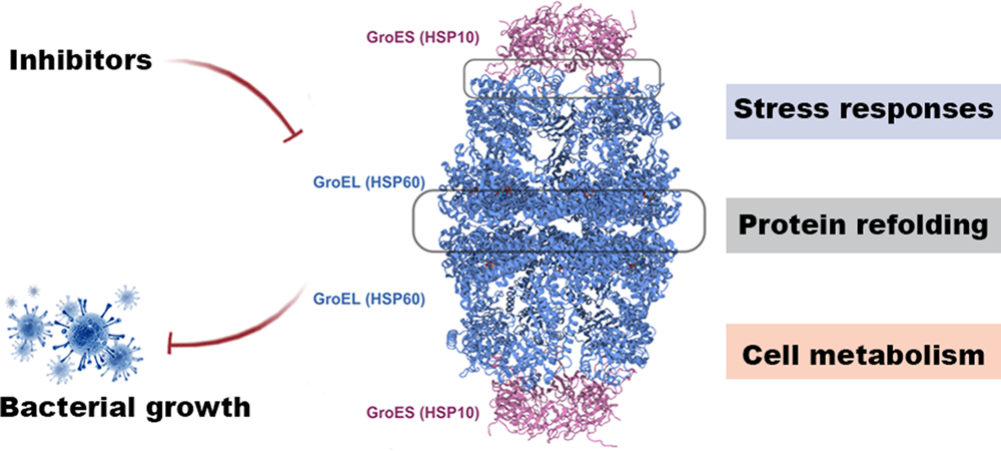

Infectious diseases have affected 13.7 million patients, placing a heavy burden on society. Furthermore, inappropriate and unrequited utilization of antibiotics has led to antimicrobial resistance worldwide. However, well-established targeted screening of environmental isolates or compound libraries has produced limited new drugs. The current situation, in which drug development is delayed, bacterial evolution is occurring, and drug resistance is emerging, requires the development of new targets and/or new strategies to combat infections. Some novel antibacterial strategies have been proposed, among which disruption of protein balance by inhibiting transcription and translation machinery is one of the proven effective antimicrobial strategies. Molecular chaperonins could mediate the correct folding of proteins, especially under conditions such as high temperature and pressure. The GroEL/ES system has been confirmed as one of the key molecular chaperones for bacterial viability. Recent data have revealed the antibacterial activities of GroEL/ES-targeted compounds, highlighting the potential role of GroEL/ES in the development of novel antibiotics. In this brief review, we discuss the function of the GroEL/ES system and summarize the inhibitors of the GroEL/ES system. The GroEL/ES system may represent a promising drug target for the exploration of novel antibiotics.

The number of deaths caused by infections was approximately 13.7 million in 2019, and reducing the burden of infection-related death is undoubtedly an urgent global public health priority.^1^ Recent research has provided comprehensive estimates of deaths associated with 33 bacterial pathogens across 11 major infectious syndromes, helping to understand the global burden of common bacterial pathogens. In this report, researchers found that the top 5 pathogens (*Staphylococcus aureus*, *Escherichia coli*, *Streptococcus pneumoniae*, *Klebsiella pneumoniae*, and *Pseudomonas aeruginosa*) were responsible for 54.9% of deaths among the bacteria studied. Among these five types of bacteria, *S. aureus* caused the most deaths in 135 countries and was also associated with the most deaths in people over 15 years old worldwide.^2^

In addition to treating infectious diseases, antibiotics have made many modern medical procedures possible, including cancer treatment, organ transplantation, and open-heart surgery. However, misuse of these valuable compounds has led to the rapid rise of antimicrobial resistance, with some infections now being untreatable.^3^ An O’Neill report commissioned by the UK government predicted that 10 million people a year will die from drug-resistant infections by 2050 unless urgent action is taken.^4^ The drying up of antibiotic discovery and the resulting unchecked spread of resistant pathogens have brought us to the brink of an antimicrobial resistance crisis.

Since 1910, various screening and chemical synthesis methods have been used to develop antibiotics, including the famous Waksman platform. However, antibiotics discovered by these methods have encountered difficulties such as the development of drug resistance of bacteria and the evolution of the bacterial cell envelope to prevent toxic compounds from entering the cell.^5−9^

Mechanistic studies showed that early antibiotics acted primarily on cellular processes, including inhibition of the enzymes involved in the synthesis of the cell wall and proteins, disruption of nucleic acid synthesis, and inhibition of protein degradation and direct damage to DNA.^10^ Accordingly, early synthetic antibiotics were designed to act in these ways. However, antibiotics designed with the idea of inhibiting cellular processes will almost certainly lead antibiotic producers (e.g., the Waksman platform, the discovery that most antibiotics use *Streptomyces* from soil) to evolve various defense mechanisms to avoid damaging themselves. The majority (nearly 60%) of antibiotics approved by the US Food and Drug Administration for clinical use over the past 30 years have been micro-natural products or derivatives thereof.^11^ Bacteria have evolved over billions of years to resist the action of natural antibacterial products, making antibiotic resistance almost a natural phenomenon. The focus of the clinical demand for effective antibiotics has therefore shifted to the need for those that can defeat multidrug-resistant organisms. The mechanism by which bacteria are either intrinsically resistant or acquire resistance to antibiotics can summarized as (1) preventing the access of antibiotics to drug targets, (2) altering the structure of antibiotic targets, and (3) directly modifying or inactivating antibiotics.^12^ As a result, the design of novel therapeutic antibiotics that can circumvent the resistance mechanism has become a research and development priority in the so-called post-antibiotic era.

## The GroEL/ES system

The concept of molecular chaperones was first introduced by Laskey. These proteins must be present in the presence of a nucleoplasm, an acidic protein in the nucleus, for the two to assemble into nucleosomes; otherwise, precipitation occurs. This class of proteins is, therefore, called molecular chaperones. Molecular chaperones can bind and stabilize the unstable conformation of another protein and facilitate the folding of nascent polypeptide chains, multimer assembly or degradation, and transmembrane transport of organelle proteins through binding and release.^13^ Chaperonins form a group of evolutionarily conserved proteins consisting of subunits with a molecular weight of ∼55 kDa. These proteins further form 800–1000 kDa double-ring complexes with 7–9 subunits per ring.^14^ Although structurally different, all proteins with this characterization can be called molecular chaperones. Heat shock proteins (HSPs) are a class of molecular chaperones whose levels are upregulated under stress.^15^ HSPs are usually named according to their molecular weight (HSP110, HSP90, HSP70, HSP60, and small HSPs).^16^ In bacteria, GroEL/ES, which is the most prominent chaperonin, is closely related to HSP60 and HSP10 in the mitochondria of eukaryotic cells.^17,18^

GroEL is a product encoded by the *groEL* gene from *E. coli*. This gene encodes a polypeptide of 548 amino acids and is also part of the gore operon, which contains the *groES* (growth essential small) gene for GroES (cochaperonin for GroEL, which consists of 97 amino acids).^19^ The crystal structure of GroEL shows a porous cylinder of 14 subunits composed of two seven-membered rings stacked back-to-back, with each subunit in one ring contacting two subunits in the opposite ring, which has a dyadic symmetry. The structure consists of three domains: (1) the equatorial ATP-binding domain (residues 6–133 and 409–523) at the waist, which forms the base of the assembly and holds the rings together; (2) the loosely structured apical domain (residues 191–376), which forms the ends of the cylinder and binds non-native substrate protein (SP) and GroES; and (3) a small slender intermediate domain (residues 134–190 and 377–408), which connects the two and create side windows.^20^ The equatorial domain provides all the intra- and inter-ring contacts, while the apical domain forms the entrance to the ring cavity and is connected to the equatorial domains via the intermediate domain, which transmits conformational changes triggered by nucleotide binding and hydrolysis.^21^ The apical domain exposes multiple hydrophobic amino acids toward the ring center, forming a circular surface for the binding of a non-native SP. The structure of GroES is a dome-shaped heptameric ring of seven 10 kDa subunits, approximately 75 Å in diameter and 30 Å high, with an 8 Å orifice in the center of its roof.^22^ Upon binding ATP and GroES, GroEL could function as a nanocage for the folding of single protein molecules to occur in isolation, unimpaired by aggregation.^23^ The binding of GroEL and GroES has therefore been described as putting a lid on protein folding.^24^

The GroEL/ES system is involved in the folding of ∼10% of the *E. coli* proteome, including those proteins that cannot use the upstream chaperones for folding.^25,26^ The GroEL-mediated refolding process typically involves the binding of GroES to the apical domain of GroEL, which encapsulates the substrate polypeptides within the central cavity of GroEL. This encapsulation isolates the polypeptides from the external environment, providing a protected space where they can attempt to fold properly.^21^ In the first step of the reaction cycle, the non-native SP binds to the free end of the GroEL/GroES complex, and then the ATP-mediated conformational rearrangement of each subunit of GroEL and the binding of GroES will lead to the encapsulation. In the nanocage formed by the chaperonin, folding occurs on each subunit of the heptameric ring for the time required for ATP hydrolysis (∼2–10 s). The protein folding cycle of the chaperonin system is complete until the binding of ATP to the opposite ring triggers the release of GroES and the folded proteins. If the folding of the proteins is not completed, it will rebind after release.^27−29^ In addition to ensuring proper protein folding, chaperonins can significantly accelerate the folding of some of these proteins by smoothing their folding energy landscape.^30−32^

The mechanism of protein folding mediated by GroEL/ES is still unclear, and much early stage research has focused on kinetic studies as well as the combined form and substructure of GroEL and GroES. Many biochemical and structural studies have suggested that the GroEL/ES system usually functions as a nanocompartment for single protein molecules to fold in isolation. The process of protein refolding can be succinctly described as a combination of purified GroEL and GroES with ATP or ADP, along with reaction substrates for subsequent detection (including chromatography and fluorescence detection).^33−36^ Imaging techniques were then gradually introduced into this area of research,^37,38^ and early structural studies were often used in conjunction with computational models.^38−40^ Similarly, molecular dynamics simulations have been widely used to investigate the role of confinement in chaperonin-mediated folding.^41−44^ Pending further development of imaging techniques, the specific protein conformation of the GroEL/ES system has been reported. For example, the football-shaped functional complex GroEL:ES_2_ has been reported by CryoEM imaging.^45^ Until recently, single-molecule techniques offered insights into the mechanisms and functional properties of biomolecules at the molecular level.^46^

Chaperonins are essential for cell viability because they are required for the efficient folding of numerous proteins by mediating the formation of the native conformation of proteins, first by preventing folding during synthesis or membrane translocation and subsequently through mediating the stepwise ATP-dependent release that leads to proper folding.^47^ In addition, the diversity of biological functions of GroEL/ES also makes this system crucial. As the study of GroEL/ES progresses, the major paradox is that this type of protein exhibits multiple biological activities that are not related to protein folding. The additional biological activities of proteins are known as moonlighting activities, and accordingly these types of proteins are defined as moonlighting proteins.^48^ So far, the number of proteins with moonlighting activities is limited. However, none of them have been observed to have such a diverse range of moonlighting activities as the GroEL protein. The moonlighting activities of GroEL reported so far include a toxin that paralyzes cockroaches,^49^ binding to cell surfaces and activating signaling pathway as an adhesin,^50−54^ binding to invertase (a hyperglycosylated mannoprotein from *Saccharomyces* cerevisiae),^55^ and binding to DNA with low specificity but high affinity.^56^

Since the production of the HSPs and the GroEL/ES system is enhanced under stress conditions, many researchers have focused on investigating the function of the system under stressful environments. Studies have indicated that the overproduction of GroEL/ES protects against high temperatures,^57^ and the system is remarkably temperature stable with an unfolding temperature above 70 °C.^58^ Moreover, although the stability of the system is much lower than that of monomeric proteins (up to ∼5 kbar for GroES), the structural integrity of the system is maintained in a relatively narrow pressure range (from ∼1 to 1000 bar), which is exactly the pressure range encountered by life on Earth.^58^ Not only that, metatranscriptomics in 2022 confirmed that the potential contribution of heat shock protein gene expression to drought-enriched taxa in extreme summer.^59^ The stabilizing effect of GroEL/ES coexpression on protein was also confirmed in an animal experiment.^60^

In terms of physiological function, the GroEL/ES system is undoubtedly essential for bacteria survival,^61^ and it can be actively secreted by both Gram-positive (G^+^) and Gram-negative (G^–^) bacteria. Several gene-level studies have also demonstrated the essential role of GroEL/ES in survival on GroEL/ES-deficient bacteria.^62,63^ Mutations in *groEL* and *groES* genes will block the growth of bacteriophages λ and T4.^64^ Studies have shown that some diseases related to bacterial infections are also associated with the GroEL/ES system. The GroEL/ES system can act as an extracellular signaling molecule and thereby influences the immune system of the host.^50,65^ Research indicated that after 24–48 h of *C. pneumoniae* infection in the HepG2 cells, the protein expression of GroEL/ES increased more than 2-fold during the redifferentiation from the reticular body to the elementary body.^66^ In another study, a series of GroEL/ES inhibitors were identified that could inhibit *M. tuberculosis* growth and the biochemical function of the protein tyrosine phosphatase B (a virulent factor secreted by *M. tuberculosis*). After optimization, this type of inhibitor can be effective against all stages of tuberculosis.^67^

## Inhibitors of the GroEL/ES System

**Figure 1:**
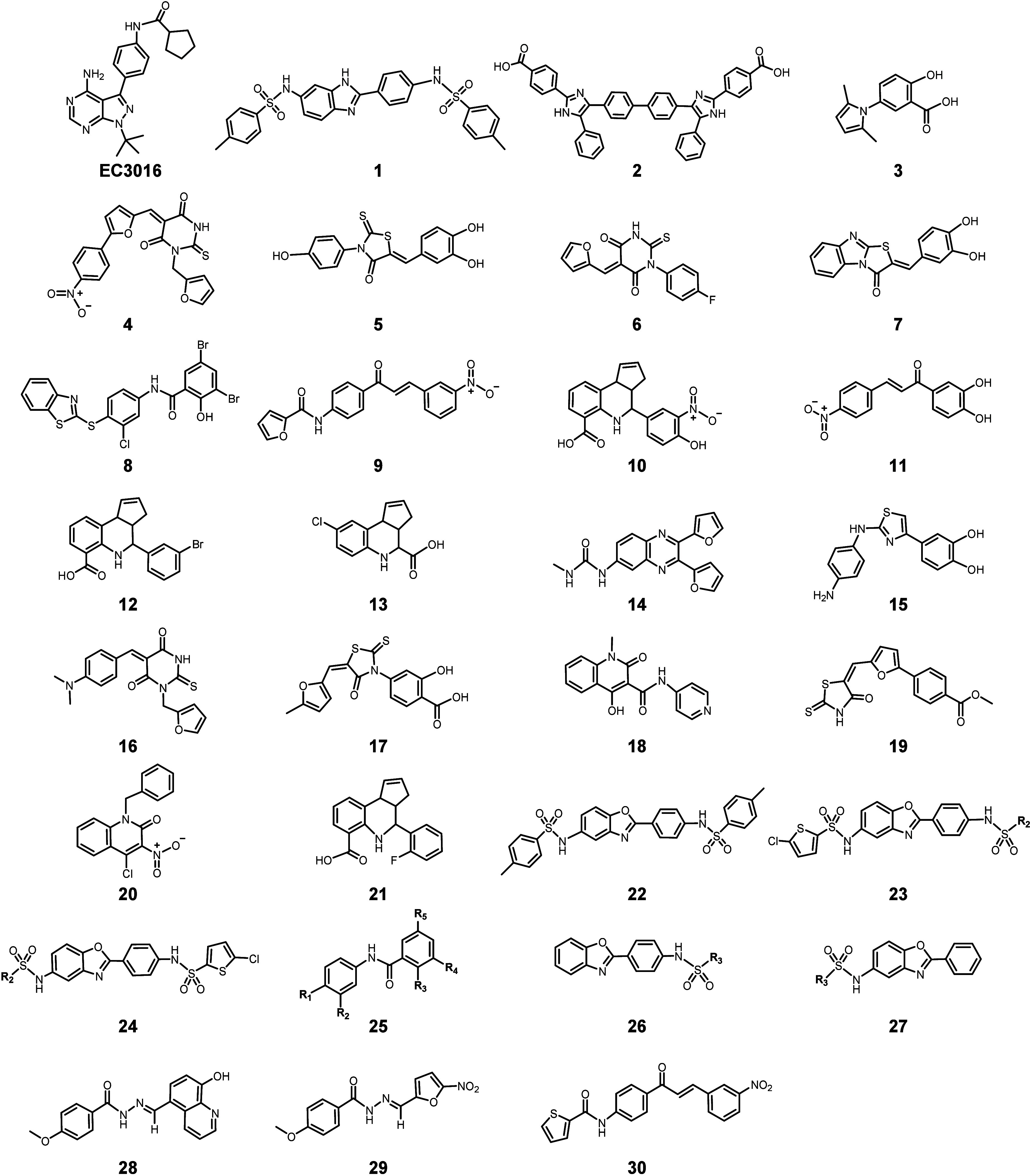
Chemical structures of the known GroEL/ES system inhibitors. Since 2014, Johnson’s group has been working on the development of GroEL/GroES inhibitors, and a series of products have been determined by chemical synthesis and biological activity evaluation of their antibacterial, antiparasitic, and antitumor capabilities.

Although antibiotic research and development seems to be reaching a bottleneck, GroEL/ES inhibitors are potential new antibiotics. Since 2014, research on GroEL/ES inhibitors has been carried out by Johnson’s group. They have mainly synthesized 3 series of compounds based on the structure of the active groups (nitroxoline, nifuroxazide, and the core scaffold sulfonamido-2-arylbenzoxazole in compound **1**) (Figure 1). These compounds were also evaluated for their antibacterial, antiparasitic, and antitumor properties.

Chapman et al.^68^ first designed and synthesized active pyrazolo-pyrimidine scaffold compounds as kinase-directed inhibitors and screened them against a collective of GroEL nucleotide pocket variants to identify a cyclopentyl carboxamide derivative, EC3016, which specifically inhibited ATPase activity and protein folding by the GroEL mutant, I493C. This orthogonal pair facilitated the action of ATP in triggering the activation of GroEL-mediated protein folding and further studies of GroEL action *in vivo*. The first 21 compounds (Figure 1, **1**–**21**) were discovered by Eli Chapman’s group in a high-throughput screen of 700 000 small molecules. These compounds were shown to be potent inhibitors of GroEL/ES-mediated refolding (IC_50_ < 10 μM).^69^ The primary assay, using the enzyme β- arylsulfotransferase-IV as a chaperonin substrate, laid the groundwork for subsequent research by this group. Further research indicated that the GroEL/ES inhibitors had antibacterial activity and were more effective at inhibiting the proliferation of G^+^ bacteria, particularly *S. aureus*. Two lead compounds **8** and **18** (Figure 1) exhibited antibiotic effects in the low-μM to mid-nM range (from 130 nM to 30 μM).^70^ After further medicinal chemistry optimization, the therapeutic windows of compound **22** were broadened.^71^ Research indicates that compounds **1** and **22** have treatment effect (EC_50_ = 7.9 and 3.1 μM) for Africa sleeping sickness (caused by the *T. brucei* infection), indicating that the strategy of GroEL/ES inhibition for potential novel antibiotics is rational. Furthermore, Abdeen et al. designed a series of asymmetric analogs based on a pseudosymmetrical bis-sulfonamido-2-phenylbenzoxazole scaffold and the structure of compound **1**. The pertinent activity assay demonstrated that these synthetics exert an inhibitory effect on the proliferation of *E. faecium* and *S. aureus*, with an EC_50_ of 1–2 μM.^72^ In addition, the benzothiazole and hydroxyl groups were found to be instrumental in the inhibition of GroEL/ES-mediated folding functions, while the hydroxyl group is essential for the antibacterial effects. The structure–activity relationship of compound **8** also led to compound **25** and its analogues, which further revealed that this series of GroEL/ES inhibitors are active against planktonic and biofilm forms to inhibit *S. aureus*.^73^ The results of this research indicated that even the GroEL/ES inhibitors were largely ineffective against *K. pneumoniae*, *A. baumannii*, *P. aeruginosa*, and *E. cloacae*, although many were potent inhibitors of *E. faecium* and *S. aureus* proliferation. Subsequently, a series of dual-targeting GroEL/ES and PtpB inhibitors, compounds **26** and **27**, were identified, which demonstrated the ability to inhibit the growth of *M. tuberculosis* and also to inhibit the biochemical biological function of PtpB.^74^ Based on the results of the structure–activity relationship (SAR) study, two series of analogues with key substructures similar to nitroxoline (hydroxyquinoline moiety, compound **28**) and nifuroxazide (biscyclic-*N*-acylhydrazone scaffolds, compound **29**) were synthesized. The results of the GroEL/ES-mediated substrate refolding assay revealed that only the metabolites of the nitrofuran-bearing analogues exhibited the greatest inhibitory potential as pro-drugs, with the ability to inhibit GroEL/ES.^75^ As mentioned above, GroEL/ES is the bacterial homologue of human HSP60/10. Ray et al. developed a series of bis-aryl-α,β-unsaturated ketone (ABK) HSP60 inhibitor analogues (compound **30**) and found 24 inhibitors that exhibited potent selectivity for targeting colorectal cancer.^76,77^

**Figure 2:**
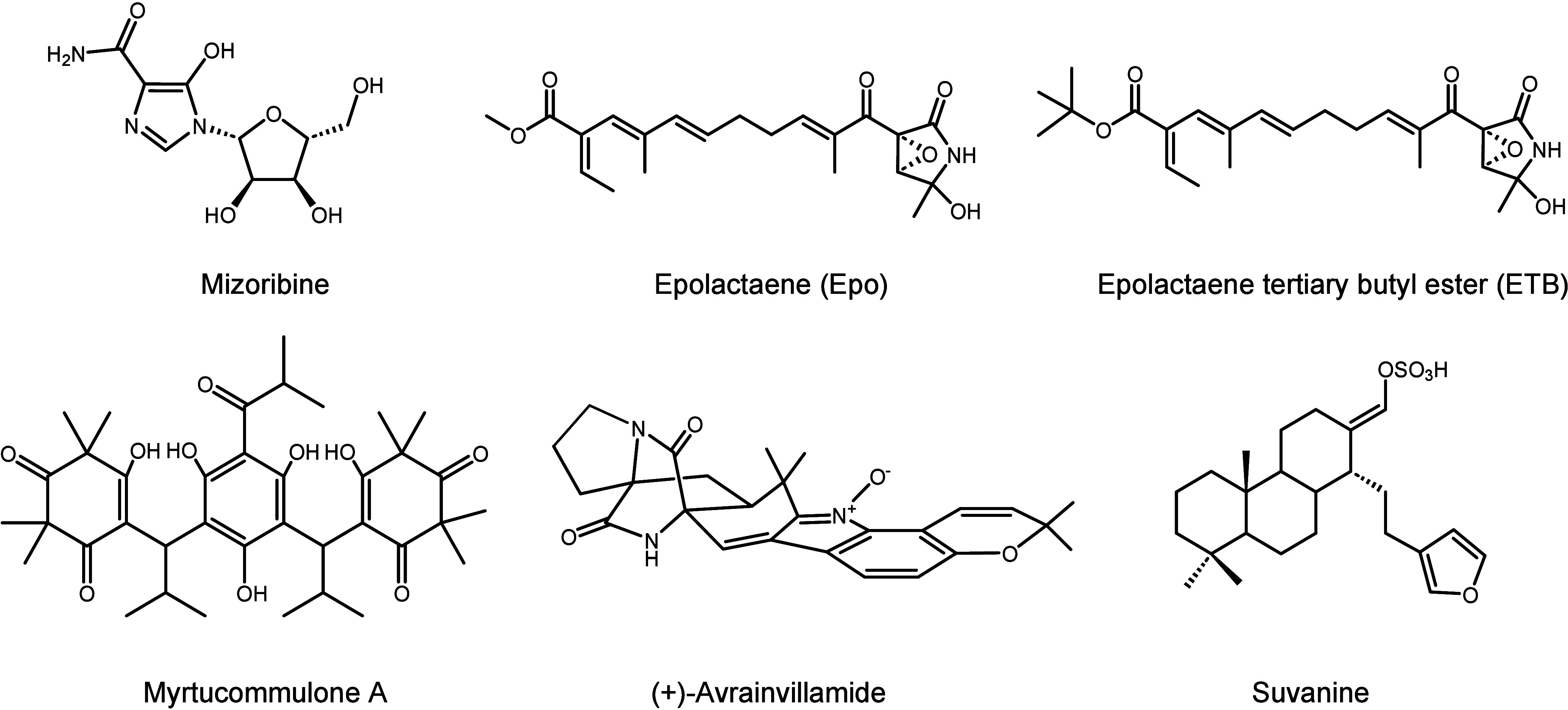
Chemical structures of the known HSP60/10 inhibitors. Six natural products show an inhibitory effect on HSP60/10, as well as antitumor, antibacterial, and anti-inflammatory activity.

**Figure 3:**
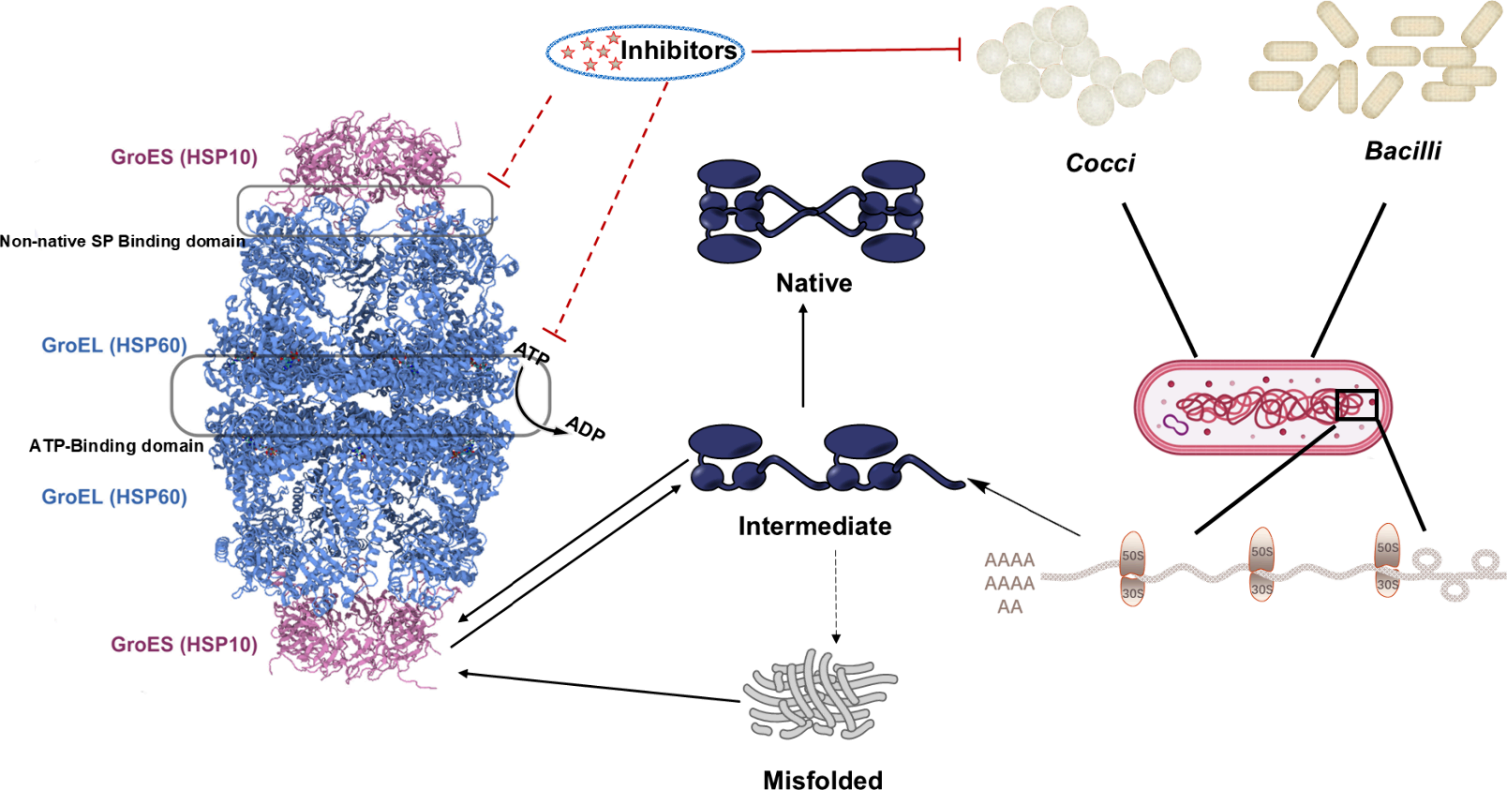
Mechanistic pathway of GroEL/ES (HSP60/10) inhibitors. The structure of HSP60/10 from eukaryotic origin is similar to that of GroEL/ES from bacteria cells. The system mediates the correct folding of proteins in an ATP-dependent form on misfolded proteins or protein intermediates as indicated by the black arrows. The inhibitors of the GroEL/ES system are supposed to have an inhibitory effect on the binding of ATP or the non-native SP on the corresponding domain, therefore showing the antibacterial effect (shown as the red arrow).

In fact, prior to the synthesis and the development of new compounds, a high-throughput screening of 3680 approved drugs and natural products identified 29 selective GroEL/ES inhibitors that showed the same inhibitory effect on the human HSP60/10 system.^70^ Unlike GroEL/ES, HSP60 serves as a mitochondrial molecular chaperone in eukaryotes, participating in the correct folding of nascent polypeptides into native proteins imported into mitochondria and stabilizing proteins to prevent excessive protein aggregation.^78^ During the development of the inhibitors of GroEL/ES, suramin, closantel, and rafoxanide were found to have inhibitory activity in both GroEL/ES and the HSP60/10 system.^79^ This finding provided a new idea that the molecules with inhibitory activity against eukaryotic HSP60/10 might also have similar inhibitory activity in the GroEL/ES system.^18^ Therefore, we have further summarized the HSP60 inhibitors in Figure 2. Mechanically, the HSP60 inhibitors mainly block the binding or hydrolysis of ATP on HSP60 while also targeting cysteine residues and other binding sites on HSP60.^80,81^ As shown, the immunosuppressant mizoribine has been confirmed to have a high affinity for HSP60 and can form a complex with HSP60, which may further affect the hydrolysis cycle of ATP in HSP60.^82^ A natural product, epolactaene, and *tert*-butyl ester ETB derived from *Penicillium* sp. showed an inhibitory effect on HSP60/10 activity, which was confirmed by protein mass spectrometry and a competition experiment. ETB was the active compound that selectively bound to HSP60 rather than HSP70 or HSP90. Furthermore, biochemical studies identified that ETB could covalently interact with cys442 of HSP60, which is located close to the ATP binding pocket.^83−85^ Similarly, avrainvillamide isolated from *Aspergillus* sp. CNC358 was found to interact with a variety of cysteine-containing proteins and showed HSP60 inhibitory activity in a Western blot assay, probably through alkylation of HSP60s cysteine residues.^86,87^ Myrtucommulone A, also a natural product with a nonprenylated acylphloroglucinol scaffold isolated from myrtle, has been reported to have antibacterial, anti-inflammatory, and antitumor properties. It directly targets the mitochondrial chaperonin HSP60 with the IC_50_ of 30 μM.^88−90^ In contrast, two HSP60-regulated proteins LONP and LRP130 appear to be protected against heat-stress-induced aggregation.^85,91^ Suvanine was a sesquiterpene natural product derived from marine sponge *Coscinoderma mathewsi*, which showed strong interaction with HSP60 through SPR and pull-down assays.^92^ We summarize the inhibitors of GroEL/ES with biological activity in Table 1 and Table 2 based on the different sources of compounds. We have also summarized the possible mechanism of the inhibitors and the protein refolding process mediated by GroEL/ES in Figure 3, taking into account the mode of action of the known inhibitors and the physiological and structural properties of the GroEL/ES.

**Table 1:**
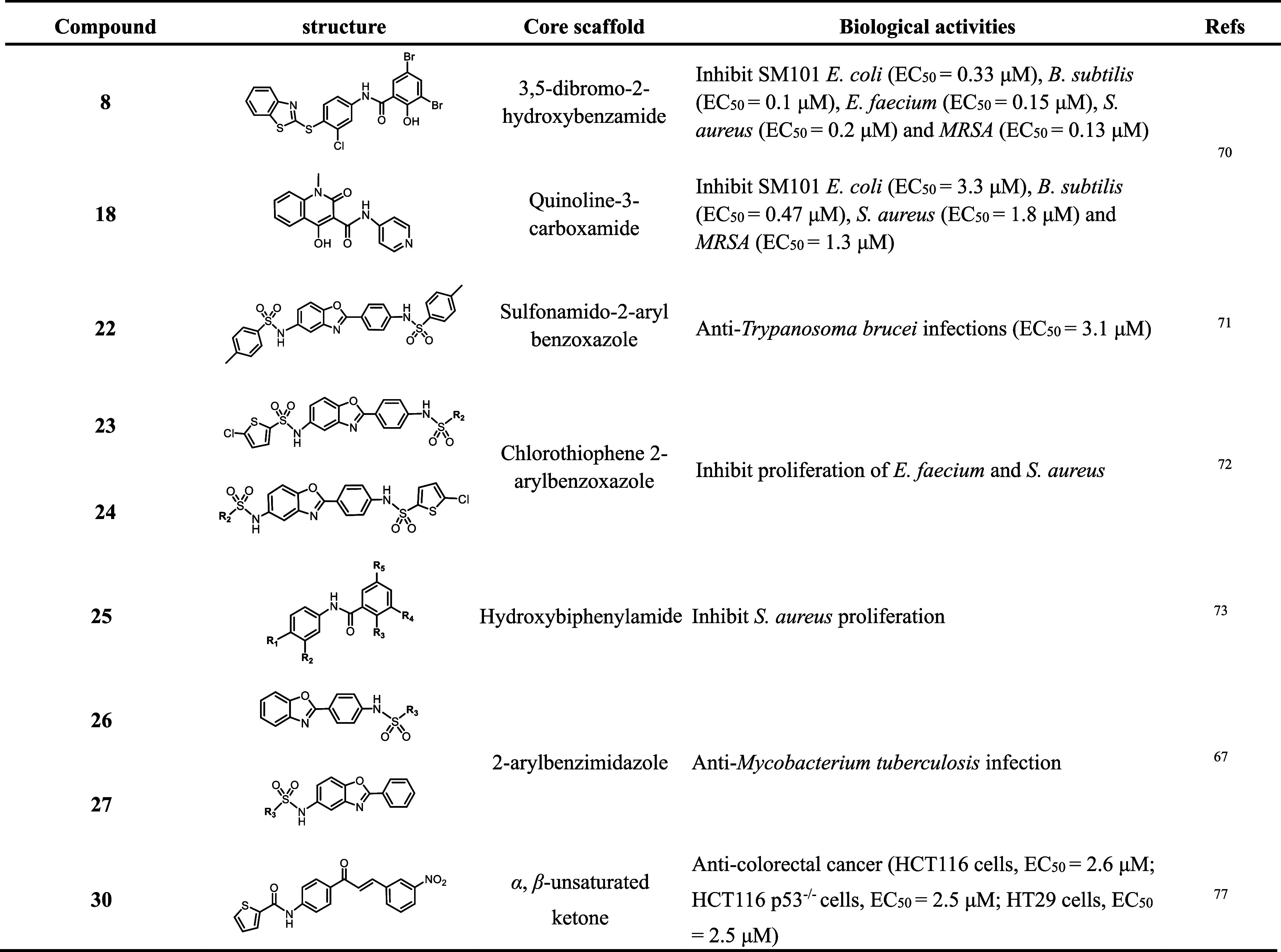
Synthetic Inhibitors of the GroEL/ES System

**Table 2:**
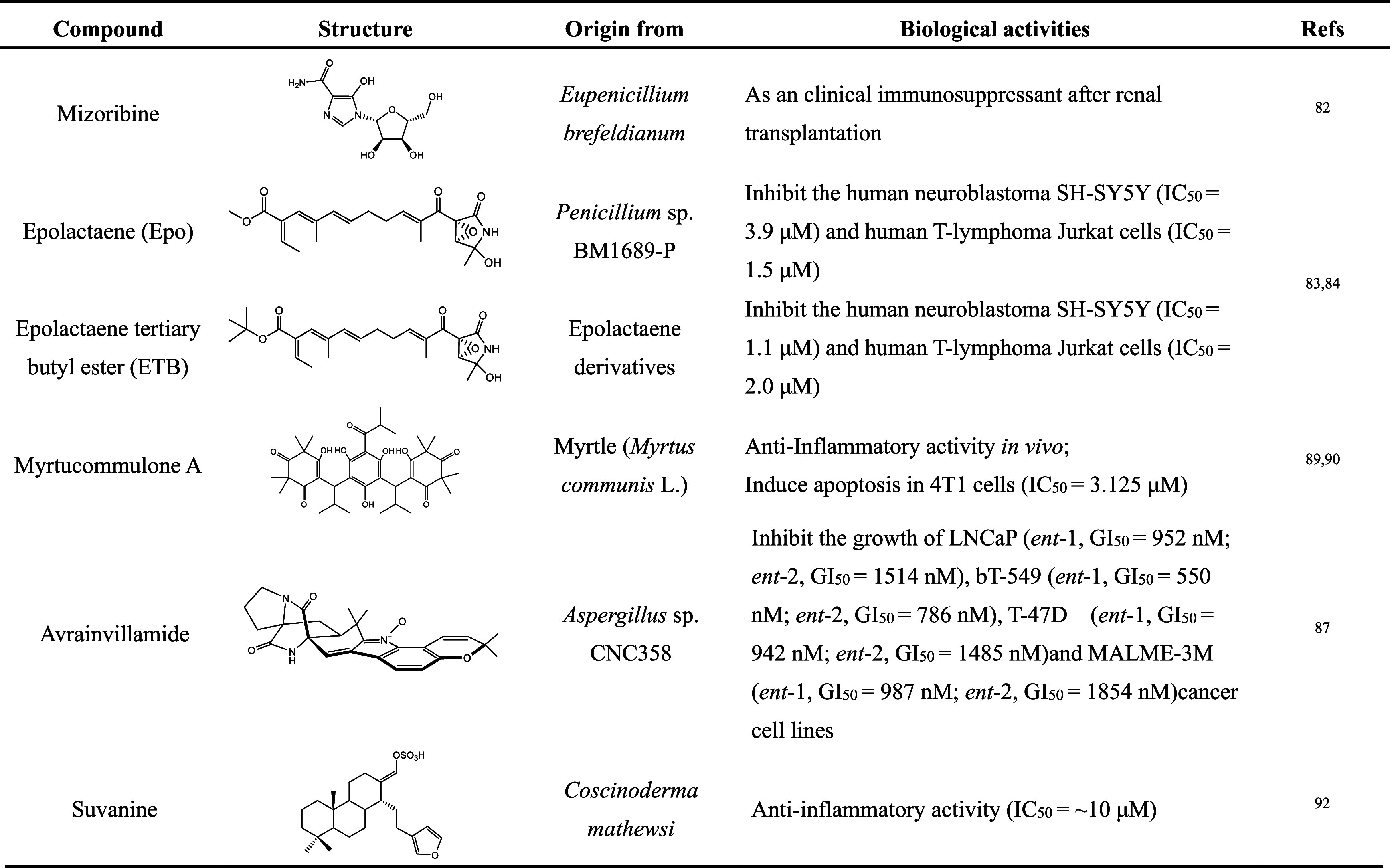
Inhibitors of GroEL/ES from Natural Products and Derivatives

## 2 Perspective and Discussion

In conclusion, the GroEL/ES system has the potential to facilitate correct protein refolding, the response to stress, and the maintenance of cellular homeostasis in bacteria. Consequently, the inhibitors of this system have demonstrated efficacy in inhibiting bacterial growth, which also suggests that the inhibitors of the GroEL/ES system are potentially effective targets for antibiotic development.

In addition, pathogens continued to accumulate resistance to existing antibiotics, and the resistance comes in many forms: limited penetrations, efflux, target modification, antibiotic destruction/modification, target switching, and sequestration.^93,94^ A model using kinetic flux theory to quantitatively predict resistance phenotypes from a unique combination of *in vitro* protein molecular properties indicated that the GroEL/ES chaperonins can affect the intracellular steady-state concentration of dihydrofolate reductase in a mutation-specific manner.^95^ The result indicated that the GroEL/ES mutant can moderate the resistance by moderating the protein stability. Besides, the RNA sequence result revealed that the *groEL/ES* gene expression improves the adaptation of pathogens to sublethal acidic conditions, resulting in increased pathogen virulence and viability.^96^ Antibiotic tolerance is also primarily responsible for the recalcitrance of chronic infections. The main approach to antimicrobial resistance is to introduce more antibiotics right now. Based on this situation, targeting the chaperonin-mediated folding cycle is a potential strategy for the resistance.

The investigation of GroEL/ES system inhibitors revealed that the benzothiazole and hydroxyl groups are crucial for inhibiting GroEL/ES-mediated folding functions, while the hydroxyl group is necessary for antibacterial effects in the derivatization synthesis based on compound **1** (Figure 1). In the development of antibacterial drugs targeting GroEL/ES, a series of compounds such as the pyrazolo-pyrimidine scaffold, the pseudosymmetric bis-sulfonamido-2-phenylbenzoxazole scaffold, or chemically synthesized compounds based on compound **1**, nitroxoline, and nifuroxazide derivatives have shown strong inhibitory activity against GroEL/ES, which may provide an ideal basis for research on antibacterial active agents.

Though extensive studies have indicated that the GroEL/ES system could be an ideal and promising target for antibiotic development, two main questions must be addressed. First, many of the studies we summarized here on the inhibitors of the GroEL/ES system have only been at the level of efficacy but have not delved into specific mechanisms related to the system. There are several reasons for this. On the one hand, as research on the mechanism of GroEL/ES-mediated protein refolding progresses, more and more substructural details of the GroEL/ES system are being revealed. This means that even when designing simple turbidity studies and refolding kinetics studies, the proportions of the substrates to GroEL/ES and the proportions of the GroEL to GroES may have an uncertain influence on the assessment of inhibitor effects. In addition, it has been shown that the ATP drives the chaperonin complex through a cycle of three functional states,^97^ which means that the rate of ATP turnover and the dwell time of GroES on the complex also need to be taken into account. The immaturity of the detection methods and the massive destabilization in the GroEL chaperonin cage made the GroEL/ES inhibitors lack merit to be an available preclinical drug.^98^

On the other hand, since the GroEL/ES is moderately conserved with the HSP60/10, inhibitors of HSP60/10 may also function as potential inhibitors of GroEL/ES. Therefore, HSP60/10 inhibitors can also be used as potential antibacterial agents in drug screening and development. The HSP60 inhibitors mainly block the binding or hydrolysis of ATP on HSP60 (mizoribine) and some target cysteine residues (ETB, avrainvillamide) and other binding sites on HSP60. The binding mode of HSP60/10 and inhibitors indicates that the latter can inhibit the activity of the formers by binding directly to HSP60, or indirectly by binding to the amino acids in the active pocket, thereby interfering with the ability of HSP60/10 to perform its function. In addition, the binding of inhibitors to HSP60/10 can also affect the role of ATP in the HSP60/10 system. It is therefore necessary to consider the various effects on the binding site when evaluating the inhibitory activity of the compounds. Due to the high homology between GroEL/ES and HSP60/10, the binding mode of HSP60/10 inhibitors may provide a mechanism reference for the in-depth development of GroEL/ES inhibitors.

Second, the homology of the GroEL/ES and HSP60/10 systems also raises the question of potential off-target effects against human cells. Therefore, in this strategy, the first and most important issues that need to be addressed are that while the inhibitors have been shown to target the chaperonin-mediated folding cycle, their selectivity needs to be addressed. Second, as mentioned above, GroEL has been recognized as a moonlighting protein, so whether targeting GroEL triggers unexpected side effects remains to be studied. Once these two concerns have been addressed, GroEL/ES system is expected to be a promising target for antibiotic discovery, regardless of the current scarcity of novel antibiotics and the emergence of bacterial resistance.

## References

[ref1] Global burden of 369 diseases and injuries in 204 countries and territories, 1990–2019: a systematic analysis for the Global Burden of Disease Study 2019. Lancet 2020, 396 (10258), 1204–1222. 10.1016/S0140-6736(20)30925-9. 33069326 PMC7567026

[ref2] Ikuta K. S; Swetschinski L. R; Robles Aguilar G.; Sharara F.; Mestrovic T.; Gray A. P; Davis Weaver N.; Wool E. E; Han C.; Gershberg Hayoon A.; Aali A.; Abate S. M.; Abbasi-Kangevari M.; Abbasi-Kangevari Z.; Abd-Elsalam S.; Abebe G.; Abedi A.; Abhari A. P.; Abidi H.; Aboagye R. G.; Absalan A.; Abubaker Ali H.; Acuna J. M.; Adane T. D.; Addo I. Y.; Adegboye O. A; Adnan M.; Adnani Q. E. S.; Afzal M. S.; Afzal S.; Aghdam Z. B.; Ahinkorah B. O.; Ahmad A.; Ahmad A. R.; Ahmad R.; Ahmad S.; Ahmad S.; Ahmadi S.; Ahmed A.; Ahmed H.; Ahmed J. Q.; Ahmed Rashid T.; Ajami M.; Aji B.; Akbarzadeh-Khiavi M.; Akunna C. J.; Al Hamad H.; Alahdab F.; Al-Aly Z.; Aldeyab M. A; Aleman A. V; Alhalaiqa F. A. N.; Alhassan R. K.; Ali B. A.; Ali L.; Ali S. S.; Alimohamadi Y.; Alipour V.; Alizadeh A.; Aljunid S. M.; Allel K.; Almustanyir S.; Ameyaw E. K.; Amit A. M. L; Anandavelane N.; Ancuceanu R.; Andrei C. L.; Andrei T.; Anggraini D.; Ansar A.; Anyasodor A. E.; Arabloo J.; Aravkin A. Y; Areda D.; Aripov T.; Artamonov A. A; Arulappan J.; Aruleba R. T.; Asaduzzaman M.; Ashraf T.; Athari S. S.; Atlaw D.; Attia S.; Ausloos M.; Awoke T.; Ayala Quintanilla B. P.; Ayana T. M.; Azadnajafabad S.; Azari Jafari A.; B D. B; Badar M.; Badiye A. D; Baghcheghi N.; Bagherieh S.; Baig A. A.; Banerjee I.; Barac A.; Bardhan M.; Barone-Adesi F.; Barqawi H. J.; Barrow A.; Baskaran P.; Basu S.; Batiha A.-M. M.; Bedi N.; Belete M. A.; Belgaumi U. I.; Bender R. G; Bhandari B.; Bhandari D.; Bhardwaj P.; Bhaskar S.; Bhattacharyya K.; Bhattarai S.; Bitaraf S.; Buonsenso D.; Butt Z. A; Caetano dos Santos F. L.; Cai J.; Calina D.; Camargos P.; Cámera L. A.; Cárdenas R.; Cevik M.; Chadwick J.; Charan J.; Chaurasia A.; Ching P. R; Choudhari S. G.; Chowdhury E. K.; Chowdhury F. R.; Chu D.-T.; Chukwu I. S.; Dadras O.; Dagnaw F. T.; Dai X.; Das S.; Dastiridou A.; Debela S. A.; Demisse F. W.; Demissie S.; Dereje D.; Derese M.; Desai H. D.; Dessalegn F. N.; Dessalegni S. A. A; Desye B.; Dhaduk K.; Dhimal M.; Dhingra S.; Diao N.; Diaz D.; Djalalinia S.; Dodangeh M.; Dongarwar D.; Dora B. T.; Dorostkar F.; Dsouza H. L.; Dubljanin E.; Dunachie S. J; Durojaiye O. C.; Edinur H. A.; Ejigu H. B.; Ekholuenetale M.; Ekundayo T. C.; El-Abid H.; Elhadi M.; Elmonem M. A; Emami A.; Engelbert Bain L.; Enyew D. B.; Erkhembayar R.; Eshrati B.; Etaee F.; Fagbamigbe A. F.; Falahi S.; Fallahzadeh A.; Faraon E. J. A; Fatehizadeh A.; Fekadu G.; Fernandes J. C; Ferrari A.; Fetensa G.; Filip I.; Fischer F.; Foroutan M.; Gaal P. A.; Gadanya M. A; Gaidhane A. M.; Ganesan B.; Gebrehiwot M.; Ghanbari R.; Ghasemi Nour M.; Ghashghaee A.; Gholamrezanezhad A.; Gholizadeh A.; Golechha M.; Goleij P.; Golinelli D.; Goodridge A.; Gunawardane D. A.; Guo Y.; Gupta R. D.; Gupta S.; Gupta V. B.; Gupta V. K.; Guta A.; Habibzadeh P.; Haddadi Avval A.; Halwani R.; Hanif A.; Hannan M. A.; Harapan H.; Hassan S.; Hassankhani H.; Hayat K.; Heibati B.; Heidari G.; Heidari M.; Heidari-Soureshjani R.; Herteliu C.; Heyi D. Z.; Hezam K.; Hoogar P.; Horita N.; Hossain M. M.; Hosseinzadeh M.; Hostiuc M.; Hostiuc S.; Hoveidamanesh S.; Huang J.; Hussain S.; Hussein N. R; Ibitoye S. E.; Ilesanmi O. S.; Ilic I. M; Ilic M. D; Imam M. T.; Immurana M.; Inbaraj L. R.; Iradukunda A.; Ismail N. E.; Iwu C. C D; Iwu C. J.; J L. M.; Jakovljevic M.; Jamshidi E.; Javaheri T.; Javanmardi F.; Javidnia J.; Jayapal S. K.; Jayarajah U.; Jebai R.; Jha R. P.; Joo T.; Joseph N.; Joukar F.; Jozwiak J. J.; Kacimi S. E. O.; Kadashetti V.; Kalankesh L. R; Kalhor R.; Kamal V. K.; Kandel H.; Kapoor N.; Karkhah S.; Kassa B. G.; Kassebaum N. J; Katoto P. D.; Keykhaei M.; Khajuria H.; Khan A.; Khan I. A; Khan M.; Khan M. N.; Khan M. A.; Khatatbeh M. M.; Khater M. M; Khayat Kashani H. R.; Khubchandani J.; Kim H.; Kim M. S.; Kimokoti R. W; Kissoon N.; Kochhar S.; Kompani F.; Kosen S.; Koul P. A; Koulmane Laxminarayana S. L.; Krapp Lopez F.; Krishan K.; Krishnamoorthy V.; Kulkarni V.; Kumar N.; Kurmi O. P; Kuttikkattu A.; Kyu H. H.; Lal D. K.; Lám J.; Landires I.; Lasrado S.; Lee S.-w.; Lenzi J.; Lewycka S.; Li S.; Lim S. S; Liu W.; Lodha R.; Loftus M. J; Lohiya A.; Lorenzovici L.; Lotfi M.; Mahmoodpoor A.; Mahmoud M. A.; Mahmoudi R.; Majeed A.; Majidpoor J.; Makki A.; Mamo G. A.; Manla Y.; Martorell M.; Matei C. N; McManigal B.; Mehrabi Nasab E.; Mehrotra R.; Melese A.; Mendoza-Cano O.; Menezes R. G; Mentis A.-F. A; Micha G.; Michalek I. M.; Micheletti Gomide Nogueira de Sá A. C.; Milevska Kostova N.; Mir S. A.; Mirghafourvand M.; Mirmoeeni S.; Mirrakhimov E. M; Mirza-Aghazadeh-Attari M.; Misganaw A. S.; Misganaw A.; Misra S.; Mohammadi E.; Mohammadi M.; Mohammadian-Hafshejani A.; Mohammed S.; Mohan S.; Mohseni M.; Mokdad A. H; Momtazmanesh S.; Monasta L.; Moore C. E; Moradi M.; Moradi Sarabi M.; Morrison S. D.; Motaghinejad M.; Mousavi Isfahani H.; Mousavi Khaneghah A.; Mousavi-Aghdas S. A.; Mubarik S.; Mulita F.; Mulu G. B. B; Munro S. B; Muthupandian S.; Nair T. S.; Naqvi A. A.; Narang H.; Natto Z. S; Naveed M.; Nayak B. P.; Naz S.; Negoi I.; Nejadghaderi S. A.; Neupane Kandel S.; Ngwa C. H.; Niazi R. K.; Nogueira de Sa A. T.; Noroozi N.; Nouraei H.; Nowroozi A.; Nuñez-Samudio V.; Nutor J. J.; Nzoputam C. I.; Nzoputam O. J.; Oancea B.; Obaidur R. M.; Ojha V. A.; Okekunle A. P.; Okonji O. C.; Olagunju A. T; Olusanya B. O.; Omar Bali A.; Omer E.; Otstavnov N.; Oumer B.; P A M.; Padubidri J. R.; Pakshir K.; Palicz T.; Pana A.; Pardhan S.; Paredes J. L; Parekh U.; Park E.-C.; Park S.; Pathak A.; Paudel R.; Paudel U.; Pawar S.; Pazoki Toroudi H.; Peng M.; Pensato U.; Pepito V. C. F.; Pereira M.; Peres M. F P; Perico N.; Petcu I.-R.; Piracha Z. Z.; Podder I.; Pokhrel N.; Poluru R.; Postma M. J; Pourtaheri N.; Prashant A.; Qattea I.; Rabiee M.; Rabiee N.; Radfar A.; Raeghi S.; Rafiei S.; Raghav P. R.; Rahbarnia L.; Rahimi-Movaghar V.; Rahman M.; Rahman M. A.; Rahmani A. M.; Rahmanian V.; Ram P.; Ranjha M. M. A. N.; Rao S. J; Rashidi M.-M.; Rasul A.; Ratan Z. A.; Rawaf S.; Rawassizadeh R.; Razeghinia M. S.; Redwan E. M. M.; Regasa M. T.; Remuzzi G.; Reta M. A.; Rezaei N.; Rezapour A.; Riad A.; Ripon R. K.; Rudd K. E; Saddik B.; Sadeghian S.; Saeed U.; Safaei M.; Safary A.; Safi S. Z.; Sahebazzamani M.; Sahebkar A.; Sahoo H.; Salahi S.; Salahi S.; Salari H.; Salehi S.; Samadi Kafil H.; Samy A. M; Sanadgol N.; Sankararaman S.; Sanmarchi F.; Sathian B.; Sawhney M.; Saya G. K.; Senthilkumaran S.; Seylani A.; Shah P. A; Shaikh M. A.; Shaker E.; Shakhmardanov M. Z.; Sharew M. M.; Sharifi-Razavi A.; Sharma P.; Sheikhi R. A.; Sheikhy A.; Shetty P. H; Shigematsu M.; Shin J. I.; Shirzad-Aski H.; Shivakumar K M; Shobeiri P.; Shorofi S. A.; Shrestha S.; Sibhat M. M.; Sidemo N. B.; Sikder M. K.; Silva L. M. L. R.; Singh J. A; Singh P.; Singh S.; Siraj M. S.; Siwal S. S.; Skryabin V. Y.; Skryabina A. A.; Socea B.; Solomon D. D.; Song Y.; Sreeramareddy C. T; Suleman M.; Suliankatchi Abdulkader R.; Sultana S.; Szócska M.; Tabatabaeizadeh S.-A.; Tabish M.; Taheri M.; Taki E.; Tan K.-K.; Tandukar S.; Tat N. Y; Tat V. Y; Tefera B. N.; Tefera Y. M.; Temesgen G.; Temsah M.-H.; Tharwat S.; Thiyagarajan A.; Tleyjeh I. I; Troeger C. E; Umapathi K. K.; Upadhyay E.; Valadan Tahbaz S.; Valdez P. R; Van den Eynde J.; van Doorn H. R.; Vaziri S.; Verras G.-I.; Viswanathan H.; Vo B.; Waris A.; Wassie G. T.; Wickramasinghe N. D.; Yaghoubi S.; Yahya G. A. T. Y.; Yahyazadeh Jabbari S. H.; Yigit A.; Yiğit V.; Yon D. K.; Yonemoto N.; Zahir M.; Zaman B. A.; Zaman S. B.; Zangiabadian M.; Zare I.; Zastrozhin M. S.; Zhang Z.-J.; Zheng P.; Zhong C.; Zoladl M.; Zumla A.; Hay S. I; Dolecek C.; Sartorius B.; Murray C. J L; Naghavi M. Global mortality associated with 33 bacterial pathogens in 2019: a systematic analysis for the Global Burden of Disease Study 2019. Lancet 2022, 400 (10369), 2221–2248. 10.1016/S0140-6736(22)02185-7. 36423648 PMC9763654

[ref3] Prescott J. F. The resistance tsunami, antimicrobial stewardship, and the golden age of microbiology. Vet. Microbiol. 2014, 171 (3–4), 273–278. 10.1016/j.vetmic.2014.02.035. 24646601

[ref4] Review on Antimicrobial Resistance Home Page. https://amr-review.org.

[ref5] Otten H. Domagk and the development of the sulphonamides. J. Antimicrob. Chemother. 1986, 17 (6), 689–696. 10.1093/jac/17.6.689. 3525495

[ref6] Hutchings M. I.; Truman A. W.; Wilkinson B. Antibiotics: past, present and future. Curr. Opin. Microbiol. 2019, 51, 72–80. 10.1016/j.mib.2019.10.008. 31733401

[ref7] Payne D. J.; Gwynn M. N.; Holmes D. J.; Pompliano D. L. Drugs for bad bugs: confronting the challenges of antibacterial discovery. Nat. Rev. Drug Discovery 2007, 6 (1), 29–40. 10.1038/nrd2201. 17159923

[ref8] Lewis K. The Science of Antibiotic Discovery. Cell 2020, 181 (1), 29–45. 10.1016/j.cell.2020.02.056. 32197064

[ref9] Tommasi R.; Brown D. G.; Walkup G. K.; Manchester J. I.; Miller A. A. ESKAPEing the labyrinth of antibacterial discovery. Nat. Rev. Drug Discovery 2015, 14 (8), 529–542. 10.1038/nrd4572. 26139286

[ref10] O’Neill E. C.; Schorn M.; Larson C. B.; Millán-Aguiñaga N. Targeted antibiotic discovery through biosynthesis-associated resistance determinants: target directed genome mining. Crit Rev. Microbiol 2019, 45 (3), 255–277. 10.1080/1040841X.2019.1590307. 30985219

[ref11] Newman D. J.; Cragg G. M. Natural Products as Sources of New Drugs from 1981 to 2014. J. Nat. Prod 2016, 79 (3), 629–661. 10.1021/acs.jnatprod.5b01055. 26852623

[ref12] Blair J. M. A.; Webber M. A.; Baylay A. J.; Ogbolu D. O.; Piddock L. J. V. Molecular mechanisms of antibiotic resistance. Nature Reviews Microbiology 2015, 13 (1), 42–51. 10.1038/nrmicro3380. 25435309

[ref13] Laskey R. A.; Honda B. M.; Mills A. D.; Finch J. T. Nucleosomes are assembled by an acidic protein which binds histones and transfers them to DNA. Nature 1978, 275 (5679), 416–420. 10.1038/275416a0. 692721

[ref14] Bracher A.; Hartl F. U. Chaperonins. In Encyclopedia of Biological Chemistry, 2 ed.; Lennarz W. J., Lane M. D., Eds.; Academic Press, 2013; pp 456–460. DOI: 10.1016/B978-0-12-378630-2.00080-3.

[ref15] Hartl F. U.; Bracher A.; Hayer-Hartl M. Molecular chaperones in protein folding and proteostasis. Nature 2011, 475 (7356), 324–332. 10.1038/nature10317. 21776078

[ref16] Richter K.; Haslbeck M.; Buchner J. The Heat Shock Response: Life on the Verge of Death. Mol. Cell 2010, 40 (2), 253–266. 10.1016/j.molcel.2010.10.006. 20965420

[ref17] Bukau B.; Horwich A. L. The Hsp70 and Hsp60 chaperone machines. Cell 1998, 92 (3), 351–366. 10.1016/S0092-8674(00)80928-9. 9476895

[ref18] Nakamura H.; Minegishi H. HSP60 as a drug target. Curr. Pharm. Des 2013, 19 (3), 441–451. 10.2174/138161213804143626. 22920899

[ref19] Chandrasekhar G. N.; Tilly K.; Woolford C.; Hendrix R.; Georgopoulos C. Purification and properties of the groES morphogenetic protein of Escherichia coli. J. Biol. Chem. 1986, 261 (26), 12414–12419. 10.1016/S0021-9258(18)67256-4. 3017973

[ref20] Braig K.; Otwinowski Z.; Hegde R.; Boisvert D. C.; Joachimiak A.; Horwich A. L.; Sigler P. B. The crystal structure of the bacterial chaperonln GroEL at 2.8 Å. Nature 1994, 371 (6498), 578–586. 10.1038/371578a0. 7935790

[ref21] Saibil H. R.; Fenton W. A.; Clare D. K.; Horwich A. L. Structure and allostery of the chaperonin GroEL. J. Mol. Biol. 2013, 425 (9), 1476–1487. 10.1016/j.jmb.2012.11.028. 23183375

[ref22] Hunt J. F.; Weaver A. J.; Landry S. J.; Gierasch L.; Deisenhofer J. The crystal structure of the GroES co-chaperonin at 2.8 A resolution. Nature 1996, 379 (6560), 37–45. 10.1038/379037a0. 8538739

[ref23] Hayer-Hartl M.; Bracher A.; Hartl F. U. The GroEL–GroES Chaperonin Machine: A Nano-Cage for Protein Folding. Trends Biochem. Sci. 2016, 41 (1), 62–76. 10.1016/j.tibs.2015.07.009. 26422689

[ref24] Saibil H. The lid that shapes the pot: structure and function of the chaperonin GroES. Structure 1996, 4 (1), 1–4. 10.1016/S0969-2126(96)00002-0. 8805512

[ref25] Hayer-Hartl M.; Bracher A.; Hartl F. U. The GroEL-GroES Chaperonin Machine: A Nano-Cage for Protein Folding. Trends Biochem. Sci. 2016, 41 (1), 62–76. 10.1016/j.tibs.2015.07.009. 26422689

[ref26] Clare D. K.; Vasishtan D.; Stagg S.; Quispe J.; Farr G. W.; Topf M.; Horwich A. L.; Saibil H. R. ATP-triggered conformational changes delineate substrate-binding and -folding mechanics of the GroEL chaperonin. Cell 2012, 149 (1), 113–123. 10.1016/j.cell.2012.02.047. 22445172 PMC3326522

[ref27] Balchin D.; Hayer-Hartl M.; Hartl F. U. In vivo aspects of protein folding and quality control. Science 2016, 353 (6294), aac4354 10.1126/science.aac4354. 27365453

[ref28] Hartl F. U.; Hayer-Hartl M. Molecular chaperones in the cytosol: from nascent chain to folded protein. Science 2002, 295 (5561), 1852–1858. 10.1126/science.1068408. 11884745

[ref29] Hartl F. U.; Bracher A.; Hayer-Hartl M. Molecular chaperones in protein folding and proteostasis. Nature 2011, 475 (7356), 324–332. 10.1038/nature10317. 21776078

[ref30] Gupta A. J.; Haldar S.; Miličić G.; Hartl F. U.; Hayer-Hartl M. Active cage mechanism of chaperonin-assisted protein folding demonstrated at single-molecule level. J. Mol. Biol. 2014, 426 (15), 2739–2754. 10.1016/j.jmb.2014.04.018. 24816391

[ref31] Tang Y. C.; Chang H. C.; Roeben A.; Wischnewski D.; Wischnewski N.; Kerner M. J.; Hartl F. U.; Hayer-Hartl M. Structural features of the GroEL-GroES nano-cage required for rapid folding of encapsulated protein. Cell 2006, 125 (5), 903–914. 10.1016/j.cell.2006.04.027. 16751100

[ref32] Chakraborty K.; Chatila M.; Sinha J.; Shi Q.; Poschner B. C.; Sikor M.; Jiang G.; Lamb D. C.; Hartl F. U.; Hayer-Hartl M. Chaperonin-catalyzed rescue of kinetically trapped states in protein folding. Cell 2010, 142 (1), 112–122. 10.1016/j.cell.2010.05.027. 20603018

[ref33] Mayhew M.; da Silva A. C.; Martin J.; Erdjument-Bromage H.; Tempst P.; Hartl F. U. Protein folding in the central cavity of the GroEL-GroES chaperonin complex. Nature 1996, 379 (6564), 420–426. 10.1038/379420a0. 8559246

[ref34] Weissman J. S.; Rye H. S.; Fenton W. A.; Beechem J. M.; Horwich A. L. Characterization of the active intermediate of a GroEL-GroES-mediated protein folding reaction. Cell 1996, 84 (3), 481–490. 10.1016/S0092-8674(00)81293-3. 8608602

[ref35] Hayer-Hartl M. K.; Weber F.; Hartl F. U. Mechanism of chaperonin action: GroES binding and release can drive GroEL-mediated protein folding in the absence of ATP hydrolysis. Embo j 1996, 15 (22), 6111–6121. 10.1002/j.1460-2075.1996.tb00999.x. 8947033 PMC452432

[ref36] Rye H. S.; Burston S. G.; Fenton W. A.; Beechem J. M.; Xu Z.; Sigler P. B.; Horwich A. L. Distinct actions of cis and trans ATP within the double ring of the chaperonin GroEL. Nature 1997, 388 (6644), 792–798. 10.1038/42047. 9285593

[ref37] Brinker A.; Pfeifer G.; Kerner M. J.; Naylor D. J.; Hartl F. U.; Hayer-Hartl M. Dual function of protein confinement in chaperonin-assisted protein folding. Cell 2001, 107 (2), 223–233. 10.1016/S0092-8674(01)00517-7. 11672529

[ref38] Meyer A. S.; Gillespie J. R.; Walther D.; Millet I. S.; Doniach S.; Frydman J. Closing the Folding Chamber of the Eukaryotic Chaperonin Requires the Transition State of ATP Hydrolysis. Cell 2003, 113 (3), 369–381. 10.1016/S0092-8674(03)00307-6. 12732144

[ref39] Xu Z.; Horwich A. L.; Sigler P. B. The crystal structure of the asymmetric GroEL–GroES–(ADP)7 chaperonin complex. Nature 1997, 388 (6644), 741–750. 10.1038/41944. 9285585

[ref40] Chen D.-H.; Madan D.; Weaver J.; Lin Z.; Schröder G. F.; Chiu W.; Rye H. S. Visualizing GroEL/ES in the Act of Encapsulating a Folding Protein. Cell 2013, 153 (6), 1354–1365. 10.1016/j.cell.2013.04.052. 23746846 PMC3695626

[ref41] Baumketner A.; Jewett A.; Shea J. E. Effects of Confinement in Chaperonin Assisted Protein Folding: Rate Enhancement by Decreasing the Roughness of the Folding Energy Landscape. J. Mol. Biol. 2003, 332 (3), 701–713. 10.1016/S0022-2836(03)00929-X. 12963377

[ref42] Takagi F.; Koga N.; Takada S. How protein thermodynamics and folding mechanisms are altered by the chaperonin cage: molecular simulations. Proc. Natl. Acad. Sci. U. S. A. 2003, 100 (20), 11367–11372. 10.1073/pnas.1831920100. 12947041 PMC208763

[ref43] Weber J. K.; Pande V. S. Functional understanding of solvent structure in GroEL cavity through dipole field analysis. J. Chem. Phys. 2013, 138 (16), 165101 10.1063/1.4801942. 23635172 PMC3651261

[ref44] Tian J.; Garcia A. E. Simulation Studies of Protein Folding/Unfolding Equilibrium under Polar and Nonpolar Confinement. J. Am. Chem. Soc. 2011, 133 (38), 15157–15164. 10.1021/ja2054572. 21854029

[ref45] Kim H.; Park J.; Lim S.; Jun S.-H.; Jung M.; Roh S.-H. Cryo-EM structures of GroEL:ES2 with RuBisCO visualize molecular contacts of encapsulated substrates in a double-cage chaperonin. iScience 2022, 25 (1), 103704 10.1016/j.isci.2021.103704. 35036883 PMC8749442

[ref46] Mistry A. C.; Chowdhury D.; Chakraborty S.; Haldar S. Elucidating the novel mechanisms of molecular chaperones by single-molecule technologies. Trends Biochem. Sci. 2024, 49, 38–51. 10.1016/j.tibs.2023.10.009. 37980187

[ref47] Ishii N. GroEL and the GroEL-GroES Complex. Subcell Biochem 2017, 83, 483–504. 10.1007/978-3-319-46503-6_17. 28271487

[ref48] Jeffery C. J. Moonlighting proteins. Trends Biochem. Sci. 1999, 24 (1), 8–11. 10.1016/S0968-0004(98)01335-8. 10087914

[ref49] Yoshida N.; Oeda K.; Watanabe E.; Mikami T.; Fukita Y.; Nishimura K.; Komai K.; Matsuda K. Protein function. Chaperonin turned insect toxin. Nature 2001, 411 (6833), 44 10.1038/35075148. 11333970

[ref50] Shin H.; Jeon J.; Lee J. H.; Jin S.; Ha U. H. Pseudomonas aeruginosa GroEL Stimulates Production of PTX3 by Activating the NF-κB Pathway and Simultaneously Downregulating MicroRNA-9. Infect. Immun. 2017, 10.1128/IAI.00935-16. PMC5328476 28031262

[ref51] Garduño R. A.; Garduño E.; Hoffman P. S. Surface-associated hsp60 chaperonin of Legionella pneumophila mediates invasion in a HeLa cell model. Infect. Immun. 1998, 66 (10), 4602–4610. 10.1128/IAI.66.10.4602-4610.1998. 9746556 PMC108567

[ref52] Pantzar M.; Teneberg S.; Lagergård T. Binding of Haemophilus ducreyi to carbohydrate receptors is mediated by the 58.5-kDa GroEL heat shock protein. Microbes Infect 2006, 8 (9–10), 2452–2458. 10.1016/j.micinf.2006.05.009. 16880000

[ref53] Bergonzelli G. E.; Granato D.; Pridmore R. D.; Marvin-Guy L. F.; Donnicola D.; Corthésy-Theulaz I. E. GroEL of Lactobacillus johnsonii La1 (NCC 533) is cell surface associated: potential role in interactions with the host and the gastric pathogen Helicobacter pylori. Infect. Immun. 2006, 74 (1), 425–434. 10.1128/IAI.74.1.425-434.2006. 16368998 PMC1346591

[ref54] Ensgraber M.; Loos M. A 66-kilodalton heat shock protein of Salmonella typhimurium is responsible for binding of the bacterium to intestinal mucus. Infect. Immun. 1992, 60 (8), 3072–3078. 10.1128/iai.60.8.3072-3078.1992. 1639475 PMC257283

[ref55] Katakura Y.; Sano R.; Hashimoto T.; Ninomiya K.; Shioya S. Lactic acid bacteria display on the cell surface cytosolic proteins that recognize yeast mannan. Appl. Microbiol. Biotechnol. 2010, 86 (1), 319–326. 10.1007/s00253-009-2295-y. 19898842

[ref56] Basu D.; Khare G.; Singh S.; Tyagi A.; Khosla S.; Mande S. C. A novel nucleoid-associated protein of Mycobacterium tuberculosis is a sequence homolog of GroEL. Nucleic Acids Res. 2009, 37 (15), 4944–4954. 10.1093/nar/gkp502. 19528065 PMC2731897

[ref57] Kandror O.; Goldberg A. L. Trigger factor is induced upon cold shock and enhances viability of Escherichia coli at low temperatures. Proc. Natl. Acad. Sci. U. S. A. 1997, 94 (10), 4978–4981. 10.1073/pnas.94.10.4978. 9144175 PMC24616

[ref58] Jaworek M. W.; Möbitz S.; Gao M.; Winter R. Stability of the chaperonin system GroEL-GroES under extreme environmental conditions. Phys. Chem. Chem. Phys. 2020, 22 (6), 3734–3743. 10.1039/C9CP06468K. 32010904

[ref59] Bei Q.; Reitz T.; Schnabel B.; Eisenhauer N.; Schädler M.; Buscot F.; Heintz-Buschart A. Extreme summers impact cropland and grassland soil microbiomes. Isme j 2023, 17 (10), 1589–1600. 10.1038/s41396-023-01470-5. 37419993 PMC10504347

[ref60] Deng J.; Li J.; Ma M.; Zhao P.; Ming F.; Lu Z.; Shi J.; Fan Q.; Liang Q.; Jia J.; Li J.; Zhang S.; Zhang L. Co-expressing GroEL–GroES, Ssa1–Sis1 and Bip–PDI chaperones for enhanced intracellular production and partial-wall breaking improved stability of porcine growth hormone. Microbial Cell Factories 2020, 19 (1), 35 10.1186/s12934-020-01304-5. 32070347 PMC7027120

[ref61] Henderson B.; Fares M. A.; Lund P. A. Chaperonin 60: a paradoxical, evolutionarily conserved protein family with multiple moonlighting functions. Biol. Rev. Camb Philos. Soc. 2013, 88 (4), 955–987. 10.1111/brv.12037. 23551966

[ref62] Chilukoti N.; Kumar C. M.; Mande S. C. GroEL2 of Mycobacterium tuberculosis Reveals the Importance of Structural Pliability in Chaperonin Function. J. Bacteriol. 2016, 198 (3), 486–497. 10.1128/JB.00844-15. 26553853 PMC4719451

[ref63] Sivinski J.; Ambrose A. J.; Panfilenko I.; Zerio C. J.; Machulis J. M.; Mollasalehi N.; Kaneko L. K.; Stevens M.; Ray A.-M.; Park Y.; Wu C.; Hoang Q. Q.; Johnson S. M.; Chapman E. Functional Differences between E. coli and ESKAPE Pathogen GroES/GroEL. mBio 2021, 10.1128/mBio.02167-20. PMC7844535 33436430

[ref64] Ang D.; Keppel F.; Klein G.; Richardson A.; Georgopoulos C. GENETIC ANALYSIS OF BACTERIOPHAGE-ENCODED COCHAPERONINS. Ann. Rev. Genet. 2000, 34, 439–456. 10.1146/annurev.genet.34.1.439. 11092834

[ref65] Friedland J. S.; Shattock R.; Remick D. G.; Griffin G. E. Mycobacterial 65-kD heat shock protein induces release of proinflammatory cytokines from human monocytic cells. Clin. Exp. Immunol. 2008, 91 (1), 58–62. 10.1111/j.1365-2249.1993.tb03354.x. PMC1554637 8419086

[ref66] Mukhopadhyay S.; Good D.; Miller R. D.; Graham J. E.; Mathews S. A.; Timms P.; Summersgill J. T. Identification of Chlamydia pneumoniae proteins in the transition from reticulate to elementary body formation. Mol. Cell Proteomics 2006, 5 (12), 2311–2318. 10.1074/mcp.M600214-MCP200. 16921167

[ref67] Washburn A.; Abdeen S.; Ovechkina Y.; Ray A.-M.; Stevens M.; Chitre S.; Sivinski J.; Park Y.; Johnson J.; Hoang Q.; Chapman E.; Parish T.; Johnson S. Dual-targeting GroEL/ES chaperonin and protein tyrosine phosphatase B (PtpB) inhibitors: A polypharmacology strategy for treating Mycobacterium tuberculosis infections. Bioorg. Med. Chem. Lett. 2019, 29, 1665–1672. 10.1016/j.bmcl.2019.04.034. 31047750 PMC6531345

[ref68] Chapman E.; Farr G. W.; Furtak K.; Horwich A. L. A small molecule inhibitor selective for a variant ATP-binding site of the chaperonin GroEL. Bioorg. Med. Chem. Lett. 2009, 19 (3), 811–813. 10.1016/j.bmcl.2008.12.015. 19110421 PMC2633924

[ref69] Johnson S. M.; Sharif O.; Mak P. A.; Wang H. T.; Engels I. H.; Brinker A.; Schultz P. G.; Horwich A. L.; Chapman E. A biochemical screen for GroEL/GroES inhibitors. Bioorg. Med. Chem. Lett. 2014, 24 (3), 786–789. 10.1016/j.bmcl.2013.12.100. 24418775

[ref70] Abdeen S.; Salim N.; Mammadova N.; Summers C. M.; Frankson R.; Ambrose A. J.; Anderson G. G.; Schultz P. G.; Horwich A. L.; Chapman E.; Johnson S. M. GroEL/ES inhibitors as potential antibiotics. Bioorg. Med. Chem. Lett. 2016, 26 (13), 3127–3134. 10.1016/j.bmcl.2016.04.089. 27184767

[ref71] Abdeen S.; Salim N.; Mammadova N.; Summers C. M.; Goldsmith-Pestana K.; McMahon-Pratt D.; Schultz P. G.; Horwich A. L.; Chapman E.; Johnson S. M. Targeting the HSP60/10 chaperonin systems of Trypanosoma brucei as a strategy for treating African sleeping sickness. Bioorg. Med. Chem. Lett. 2016, 26 (21), 5247–5253. 10.1016/j.bmcl.2016.09.051. 27720295

[ref72] Abdeen S.; Kunkle T.; Salim N.; Ray A. M.; Mammadova N.; Summers C.; Stevens M.; Ambrose A. J.; Park Y.; Schultz P. G.; Horwich A. L.; Hoang Q. Q.; Chapman E.; Johnson S. M. Sulfonamido-2-arylbenzoxazole GroEL/ES Inhibitors as Potent Antibacterials against Methicillin-Resistant Staphylococcus aureus (MRSA). J. Med. Chem. 2018, 61 (16), 7345–7357. 10.1021/acs.jmedchem.8b00989. 30060666 PMC6345161

[ref73] Kunkle T.; Abdeen S.; Salim N.; Ray A. M.; Stevens M.; Ambrose A. J.; Victorino J.; Park Y.; Hoang Q. Q.; Chapman E.; Johnson S. M. Hydroxybiphenylamide GroEL/ES Inhibitors Are Potent Antibacterials against Planktonic and Biofilm Forms of Staphylococcus aureus. J. Med. Chem. 2018, 61 (23), 10651–10664. 10.1021/acs.jmedchem.8b01293. 30392371 PMC6467803

[ref74] Washburn A.; Abdeen S.; Ovechkina Y.; Ray A. M.; Stevens M.; Chitre S.; Sivinski J.; Park Y.; Johnson J.; Hoang Q. Q.; Chapman E.; Parish T.; Johnson S. M. Dual-targeting GroEL/ES chaperonin and protein tyrosine phosphatase B (PtpB) inhibitors: A polypharmacology strategy for treating Mycobacterium tuberculosis infections. Bioorg. Med. Chem. Lett. 2019, 29 (13), 1665–1672. 10.1016/j.bmcl.2019.04.034. 31047750 PMC6531345

[ref75] Stevens M.; Howe C.; Ray A. M.; Washburn A.; Chitre S.; Sivinski J.; Park Y.; Hoang Q. Q.; Chapman E.; Johnson S. M. Analogs of nitrofuran antibiotics are potent GroEL/ES inhibitor pro-drugs. Bioorg. Med. Chem. 2020, 28 (22), 115710 10.1016/j.bmc.2020.115710. 33007545 PMC7914298

[ref76] Ray A. M.; Salim N.; Stevens M.; Chitre S.; Abdeen S.; Washburn A.; Sivinski J.; O’Hagan H. M.; Chapman E.; Johnson S. M. Exploiting the HSP60/10 chaperonin system as a chemotherapeutic target for colorectal cancer. Bioorg. Med. Chem. 2021, 40, 116129 10.1016/j.bmc.2021.116129. 33971488 PMC8194340

[ref77] Chitre S.; Ray A.-M.; Stevens M.; Doud E. H.; Liechty H.; Washburn A.; Tepper K.; Sivinski J.; O’Hagan H. M.; Georgiadis M. M.; Chapman E.; Johnson S. M. Bis-aryl-α,β-unsaturated ketone (ABK) chaperonin inhibitors exhibit selective cytotoxicity to colorectal cancer cells that correlates with levels of aberrant HSP60 in the cytosol. Bioorg. Med. Chem. 2022, 75, 117072 10.1016/j.bmc.2022.117072. 36356534 PMC11813185

[ref78] Meng Q.; Li B. X.; Xiao X. Toward Developing Chemical Modulators of Hsp60 as Potential Therapeutics. Front Mol. Biosci 2018, 5, 35 10.3389/fmolb.2018.00035. 29732373 PMC5920047

[ref79] Stevens M.; Abdeen S.; Salim N.; Ray A. M.; Washburn A.; Chitre S.; Sivinski J.; Park Y.; Hoang Q. Q.; Chapman E.; Johnson S. M. HSP60/10 chaperonin systems are inhibited by a variety of approved drugs, natural products, and known bioactive molecules. Bioorg. Med. Chem. Lett. 2019, 29 (9), 1106–1112. 10.1016/j.bmcl.2019.02.028. 30852084 PMC6450568

[ref80] Cappello F.; Marino Gammazza A.; Palumbo Piccionello A.; Campanella C.; Pace A.; Conway de Macario E.; Macario A. J. Hsp60 chaperonopathies and chaperonotherapy: targets and agents. Expert Opin Ther Targets 2014, 18 (2), 185–208. 10.1517/14728222.2014.856417. 24286280

[ref81] Taldone T.; Ochiana S. O.; Patel P. D.; Chiosis G. Selective targeting of the stress chaperome as a therapeutic strategy. Trends Pharmacol. Sci. 2014, 35 (11), 592–603. 10.1016/j.tips.2014.09.001. 25262919 PMC4254259

[ref82] Itoh H.; Komatsuda A.; Wakui H.; Miura A. B.; Tashima Y. Mammalian HSP60 Is a Major Target for an Immunosuppressant Mizoribine*. J. Biol. Chem. 1999, 274 (49), 35147–35151. 10.1074/jbc.274.49.35147. 10574997

[ref83] Kakeya H.; Takahashi I.; Okada G.; Isono K.; Osada H. Epolactaene, a novel neuritogenic compound in human neuroblastoma cells, produced by a marine fungus. J. Antibiot (Tokyo) 1995, 48 (7), 733–735. 10.7164/antibiotics.48.733. 7649877

[ref84] Nagumo Y.; Kakeya H.; Yamaguchi J.; Uno T.; Shoji M.; Hayashi Y.; Osada H. Structure-activity relationships of epolactaene derivatives: structural requirements for inhibition of Hsp60 chaperone activity. Bioorg. Med. Chem. Lett. 2004, 14 (17), 4425–4429. 10.1016/j.bmcl.2004.06.054. 15357965

[ref85] Sun W.; Wang L.; Jiang H.; Chen D.; Murchie A. I. Targeting mitochondrial transcription in fission yeast with ETB, an inhibitor of HSP60, the chaperone that binds to the mitochondrial transcription factor Mtf1. Genes Cells 2012, 17 (2), 122–131. 10.1111/j.1365-2443.2011.01578.x. 23035257

[ref86] Tabeta K.; Yamazaki K.; Hotokezaka H.; Yoshie H.; Hara K. Elevated humoral immune response to heat shock protein 60 (hsp60) family in periodontitis patients. Clin. Exp. Immunol. 2001, 120 (2), 285–293. 10.1046/j.1365-2249.2000.01216.x. PMC1905647 10792378

[ref87] Wulff J. E.; Herzon S. B.; Siegrist R.; Myers A. G. Evidence for the Rapid Conversion of Stephacidin B into the Electrophilic Monomer Avrainvillamide in Cell Culture. J. Am. Chem. Soc. 2007, 129 (16), 4898–4899. 10.1021/ja0690971. 17397160 PMC3175819

[ref88] Appendino G.; Bianchi F.; Minassi A.; Sterner O.; Ballero M.; Gibbons S. Oligomeric acylphloroglucinols from myrtle (Myrtus communis). J. Nat. Prod 2002, 65 (3), 334–338. 10.1021/np010441b. 11908974

[ref89] Rossi A.; Di Paola R.; Mazzon E.; Genovese T.; Caminiti R.; Bramanti P.; Pergola C.; Koeberle A.; Werz O.; Sautebin L.; Cuzzocrea S. Myrtucommulone from Myrtus communis exhibits potent anti-inflammatory effectiveness in vivo. J. Pharmacol Exp Ther 2009, 329 (1), 76–86. 10.1124/jpet.108.143214. 19056932

[ref90] Izgi K.; Iskender B.; Jauch J.; Sezen S.; Cakir M.; Charpentier M.; Canatan H.; Sakalar C. Myrtucommulone-A Induces both Extrinsic and Intrinsic Apoptotic Pathways in Cancer Cells. J. Biochem Mol. Toxicol 2015, 29 (9), 432–439. 10.1002/jbt.21716. 26032814

[ref91] Wiechmann K.; Müller H.; König S.; Wielsch N.; Svatoš A.; Jauch J.; Werz O. Mitochondrial Chaperonin HSP60 Is the Apoptosis-Related Target for Myrtucommulone. Cell Chemical Biology 2017, 24 (5), 614–623. 10.1016/j.chembiol.2017.04.008. 28457707

[ref92] Cassiano C.; Monti M. C.; Festa C.; Zampella A.; Riccio R.; Casapullo A. Chemical proteomics reveals heat shock protein 60 to be the main cellular target of the marine bioactive sesterterpene suvanine. Chembiochem 2012, 13 (13), 1953–1958. 10.1002/cbic.201200291. 22829539

[ref93] Alekshun M. N.; Levy S. B. Molecular Mechanisms of Antibacterial Multidrug Resistance. Cell 2007, 128 (6), 1037–1050. 10.1016/j.cell.2007.03.004. 17382878

[ref94] Lewis K. Platforms for antibiotic discovery. Nat. Rev. Drug Discovery 2013, 12 (5), 371–387. 10.1038/nrd3975. 23629505

[ref95] Rodrigues J. V.; Bershtein S.; Li A.; Lozovsky E. R.; Hartl D. L.; Shakhnovich E. I. Biophysical principles predict fitness landscapes of drug resistance. Proc. Natl. Acad. Sci. U. S. A. 2016, 113 (11), E1470-E1478 10.1073/pnas.1601441113. 26929328 PMC4801265

[ref96] Gu D.; Wang K.; Lu T.; Li L.; Jiao X. Vibrio parahaemolyticus CadC regulates acid tolerance response to enhance bacterial motility and cytotoxicity. J. Fish Dis 2021, 44 (8), 1155–1168. 10.1111/jfd.13376. 33831221 PMC8359830

[ref97] Ranson N. A.; Burston S. G.; Clarke A. R. Binding, encapsulation and ejection: substrate dynamics during a chaperonin-assisted folding reaction. J. Mol. Biol. 1997, 266 (4), 656–664. 10.1006/jmbi.1996.0815. 9102459

[ref98] Korobko I.; Mazal H.; Haran G.; Horovitz A. Measuring protein stability in the GroEL chaperonin cage reveals massive destabilization. Elife 2020, 9, e56511 10.7554/eLife.56511. 32716842 PMC7440923

